# Investment choice and perceived mating intentions regulated by external resource cues and internal fluctuation in blood glucose levels

**DOI:** 10.3389/fpsyg.2014.01523

**Published:** 2015-01-06

**Authors:** Li-Lin Rao, Xiao-Tian Wang, Shu Li

**Affiliations:** ^1^Key Laboratory of Behavioral Science, Institute of Psychology, Chinese Academy of SciencesBeijing, China; ^2^Department of Psychology, University of South DakotaVermillion, SD, USA

**Keywords:** resource allocation, blood glucose, hierarchy of needs, foraging, mating, life history

## Abstract

We examined resource allocation priorities in the framework of an updated Maslow hierarchy of fundamental human needs. In Experiment 1, the participants in the food abundance priming condition viewing photos of high-calorie food allocated more money to savings than to spending. However, the participants preferred spending to savings under the condition of mating availability priming with romantic photographs. In Experiment 2, before and after drinking either water or a sugary beverage, fasting participants rated photos of a conversation between a man and a woman. Water drinking lowered the rating scores of mating intentions as well as blood glucose (BG) levels. The sugary drink buffered this decline in sexual perceptivity. Overall, the change in BG levels was positively associated with changes in the ratings of mating intentions but was not associated with other likelihood ratings. These results suggest that both external cues of food and mating resources and internal BG fluctuation regulate the cognitive priority of physiological needs vs. mate acquisition and retention.

## Introduction

Energy, food, nutrients, calories, money, time, and mates are all resources needed for survival and reproduction. The acquisition and use of resources and of information are two major processes vital to all living organisms across the lifespan (Glazier, 2009). The relationship between foraging and mating, two major forms of resource acquisition, has been of long-standing interest across multiple disciplines. From the perspective of evolutionary biology, life is the result of a process by which variant forms of organisms compete to acquire energy from the environment and replicate (Kaplan and Gangestad, 2005). Individuals capture food energy from the environment (through foraging, hunting, or cultivating) and allocate this energy to activities that enhance survival (e.g., somatic growth, savings), mating, and reproduction. Given that individuals must live within finite energy budgets in a biological reality, adaptive decisions regarding the investment of time and energy in various life tasks relies on the cognitive priority of different human needs and motives based on external and internal resource availabilities (Kenrick et al., 2009, 2010).

Similarly, life-history models assume that resources are always limited. Organisms can be considered “informed resource users” who allocate limited resources to substantiate life-history priorities (e.g., survival, somatic growth, mating, or reproduction) based on their relative importance at a given time in life. What constitutes a favorable or unfavorable tradeoff depends on a dynamic interaction between fundamental needs or motivational systems and environmental conditions. Maslow's hierarchy of fundamental human needs and motivation (Maslow, 1943, 1954) has been widely adopted in both the biological and social sciences as a basic theoretical framework. However, although commonly assumed in more recent research, little is known about the actual tradeoffs between different needs. An updated hierarchy of human needs and motives proposed by Kenrick et al. (2010) provides a new framework to experimentally explore the tradeoffs between physiological needs for food and higher-order needs for mate acquisition and retention.

By integrating ideas from life-history theory with Maslow's classic hierarchy, Kenrick et al. (2010) suggest a natural hierarchical relationship between survival and reproductive goals (i.e., mate acquisition, mate retention, and parental care) in which survival goals underlying reproductive goals. Survival and social needs provide the foundation for acquiring mates, which in turn provides a foundation for meeting the needs of reproducing and parenting. The model assumes a continual dynamic interplay between these fundamental needs or motives. The activation of needs and goals is highly sensitive to proximate cues and environmental threats and opportunities. As noted by Deci and Ryan (2000), the term “need” has been used in various ways, but it is most closely associated with physiological deficits such as low blood sugar, which triggers hunger.

**Figure 1:**
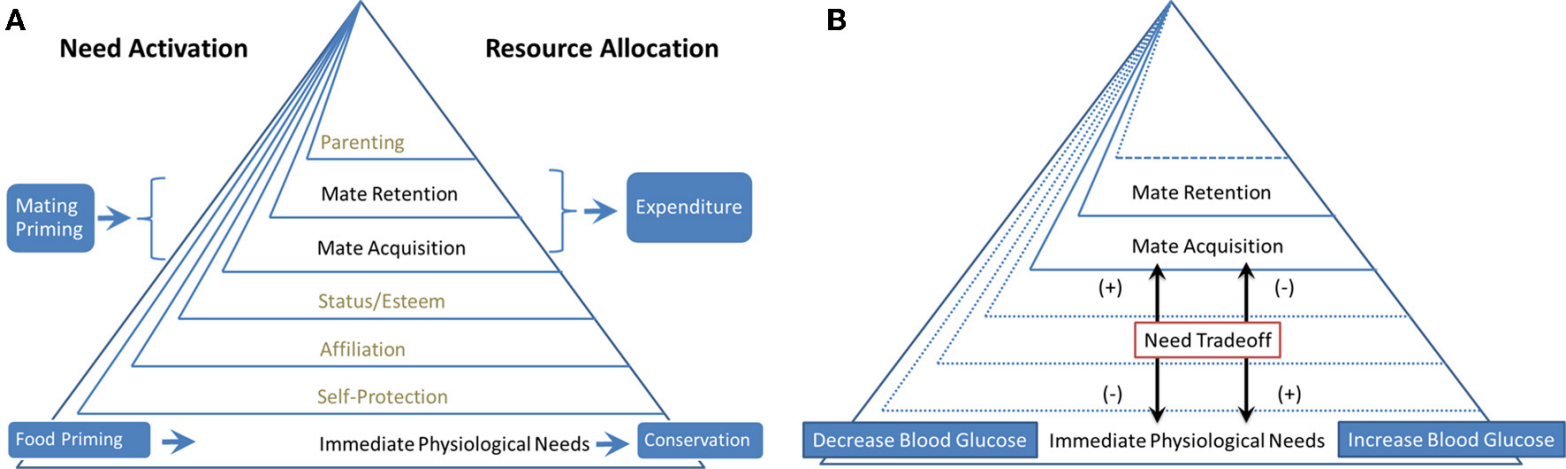
**(A)** Experiment 1. The effect of need activation on resource allocation; **(B)** Experiment 2. Regulating the cognitive priority of different needs.

Based on the hierarchy model of Kenrick et al. (2010), we propose that the tradeoff between foraging and mating priorities is regulated on a daily basis by ongoing physiological conditions, such as blood glucose (BG) levels and environmental cues, similar to the life-history tradeoffs between somatic growth and reproduction. We conducted two experiments to test this idea. The tradeoff mechanisms between hierarchical needs as manipulated in Experiments 1 and 2 are illustrated in Figures 1A,B, respectively. Experiment 1 was designed to test whether priming cues (i.e., food abundance and mating opportunities) would have different effects on monetary investment choices. Considering the natural hierarchical relationship between survival and reproductive goals, with survival goals underlying reproductive goals (Kenrick et al., 2010), we predict that priming food abundance promotes resource conservation behavior. By contrast, because mate acquisition and retention require resource expenditure (Kenrick et al., 2010), we predict that mating priming prompts resource expenditure behaviors (Figure 1). Experiment 1 examines the survival-reproduction (food-mate) priority tradeoff prompted by external cues, whereas Experiment 2 explores a similar type of food-mate tradeoff regulated by internal cues associated with BG fluctuation (Figure 1). Experiment 2 was designed to test whether changes in physiological needs manipulated by administering water or sugary drinks to fasting participants would have implicit differential effects on higher-level human motives, such as mate acquisition and retention. We hypothesize that fluctuations in BG levels signal the need for resources and regulate the acquisition priority for different resources (e.g., food or mate). When BG levels are low (even within a normal range), physiological needs predominate over mating needs. When BG levels fluctuate upward, mate acquisition and retention needs become more important (Figure 1).

## Experiment 1

We examined the differential effects of food abundance cues and mating availability cues on monetary allocation choices.

A review of the evolutionary psychology literature suggests that mating is a resource acquisition process that involves tradeoffs between the expenditure of financial resources and the acquisition of mating resources (Buss, 1994, 2005; Wilson and Daly, 2004; Li and Kenrick, 2006; Saad and Vongas, 2009; Burnham and Phelan, 2012; Griskevicius et al., 2012).

Wilson and Daly (2004) reported that priming mating opportunities with photos of attractive persons of the opposite sex changes the rate of temporal delay discounting. Photos of attractive women increased the delay discounting of men for monetary rewards. Hence, male participants were more likely to prefer a smaller and sooner reward over a larger but delayed reward. Thus, mating cues activate a spending mode of intertemporal choice. Similarly, more recent studies have also indicated that exposure to sexually provocative cues (e.g., viewing sexy photos or fondling a bra) increases both the urgency to consume something rewarding and the preference for smaller and quicker rewards (Van den Bergh et al., 2008). These findings highlight the link between monetary resource expenditure and mating cues.

In view of the experimental evidence from previous studies (e.g., Wilson and Daly, 2004), we predict that mating priming prompts spending behaviors. In addition, we predict that priming food abundance promotes saving behaviors (see also Figure 1A).

### Methods

A total of 120 participants (60 males and 60 females with an average age of 19.9 ± 0.9 years) were recruited from a large university in Beijing. The participants were assigned to one of three conditions: (a) a food priming condition that used photos of high-calorie food items (e.g., gourmet food, desserts, and fast food), (b) a mating priming condition that used romantic photos and paintings of a couple kissing or hugging one another^1^, and (c) a control condition that used abstract paintings of colors and shapes. Forty (20 female and 20 male) participants were assigned to each condition. For each condition, the participant was asked to initially view a group of five printed photos and then answer several questions regarding the photos.

In the food priming condition, each participant was asked to indicate the extent to which they agreed with one statement regarding each of the five photos on a 7-point Likert scale ranging from 1 (completely disagree) to 7 (completely agree).

The following statement was used: “This photo stimulates my appetite.”

In the mating priming condition, each participant was given the same 7-point rating scale for the following statement regarding each of the five romantic photos: “This picture is sexy.”

In the control condition, the participants were asked to indicate their agreement or disagreement on the same 7-point scale for each of the five photos of abstract paintings with respect to the following two statements: “This picture is sexy” and “This picture stimulates my appetite.”

After completing the rating task, the participants were presented with a personal financial investment scenario adapted from Liu and Aaker (2007). In this scenario, the participants allocate money for savings and for immediate spending. The scenario reads as follows.

“Please imagine you have just received a Ұ10,000 bonus from your company. What would you do with this money? Please indicate the amount you would allocate for each of the following purposes (must total Ұ10,000).

- Spend now: Ұ____;
- Put in a savings account Ұ____.”

### Results and discussion

An independent-sample *t*-test of appetite ratings revealed that individuals in the food priming group had higher appetite rating scores than the control group did, *t*_(77)_ = −6.339, *p* < 0.001. An independent-sample *t*-test of the sexiness ratings revealed that individuals in the mating priming group had higher sexiness rating scores than the control group did, *t*_(77)_ = −2.762, *p* = 0.007. These results indicated that our manipulation was effective.

**Figure 2:**
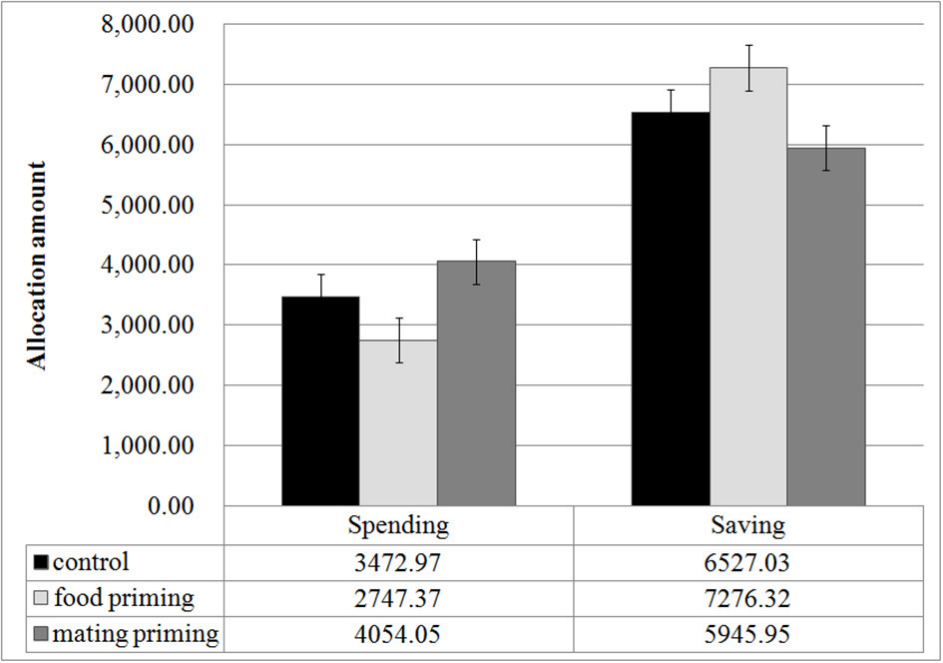
**Monetary allocation under food and mating priming conditions**. Error bars represent standard errors.

Figure 2 presents the descriptive results of Experiment 1. The gender of the participants did not affect the amount of saving, *t*_(109)_ = 0.760, *p* = 0.449, or the amount of spending, *t*_(109)_ = 0.723, *p* = 0.471. We thus pooled the data for both sexes in the following analysis. A One-Way ANOVA of the amount of allocation revealed a significant effect of priming on saving decisions, *F*_(2, 109)_ = 3.054, *p* = 0.05, η^2^ = 0.053, and spending decisions, *F*_(2, 109)_ = 3.167, *p* = 0.046, η^2^ = 0.055. The results of a Two-Way ANOVA also supported our prediction: with investment option (saving or spending) as a within-subject variable and priming as a between-subjects variable, the Two-Way ANOVA on the amount of allocation revealed a significant main effect of the investment option, *F*_(1, 109)_ = 53.005, *p* < 0.001, η^2^ = 0.327, and a significant interaction between the investment option and the priming condition, *F*_(2, 109)_ = 3.111, *p* = 0.049, η^2^ = 0.054. A *post-hoc* analysis showed that (1) the participants in the mating priming condition allocated more money to immediate spending than did those in the food priming group, *p* = 0.015, and (2) the participants in the food priming group allocated more money to savings (72.6% of the total allocation) than did those in the mating priming group (59.5% of the total allocation, *p* = 0.014).

Collectively, these results support our prediction that priming mating opportunities motivates immediate monetary spending, whereas priming food abundance promotes saving intentions and future-oriented investment.

## Experiment 2

The results from Experiment 1 suggest that investment decisions are made based on the availability of different types of resources (food or mate) as indicated by relevant external cues. In Experiment 2, we explored a similar type of food-mate priority tradeoff regulated based on internal cues of BG fluctuations (see also Figure 1).

Perrigo (1990) argued that all somatic and behavioral processes of survival and reproduction are driven by available food energy, which is in turn indexed by BG levels. One of our earlier studies (Wang and Dvorak, 2010) identified a novel link between BG levels and delay discounting in the process of making inter-temporal choices. We found that participants are more future oriented and prefer larger but delayed options when their BG levels are higher after drinking a sugary drink than after drinking a diet drink. These findings suggest that fluctuating BG levels inform the brain of the ongoing energy condition of the body and allow the brain to regulate metabolic and behavioral reactions to various demands for resource acquisition and use.

We hypothesize that fluctuations in BG levels signal the need for resources and regulate the priority of acquiring different resources (e.g., food or mate). When BG levels are low, physiological needs predominate over mating needs, as indicated in the classic Minnesota starvation experiment (Keys et al., 1950; Tucker, 2008). When BG levels fluctuate upward within a normal range, the need for mate acquisition and retention receives greater priority.

In particular, we predict that participants in a fasting state would be less sensitive to sexual cues when their BG levels further decrease after drinking water. However, compared with the water-drinking participants, participants drinking a sugary beverage would more sensitive to the same mating cues. Overall, we predict that the change (rise or fall) in BG levels will be positively correlated with changes in participants' sensitivity to mating cues (sexual perceptivity).

### Methods

A total of 134 participants (71 males and 63 females with an average age of 22.8 ± 2.2 years) were recruited from a large university in Beijing through an advertisement. All participants were informed about BG checks and provided written informed consent prior to the experiment. Their BG levels were measured using the Accu-Chek® Active Blood Glucose Meter (Roche Diagnostics GmbH, Mannheim, Germany). The height and weight of the participants were also assessed. Their body mass index (BMI), a proxy for human body fat, was subsequently calculated using the following formula: Weight (kg)/(Height (m))^2^.

**Figure 3:**
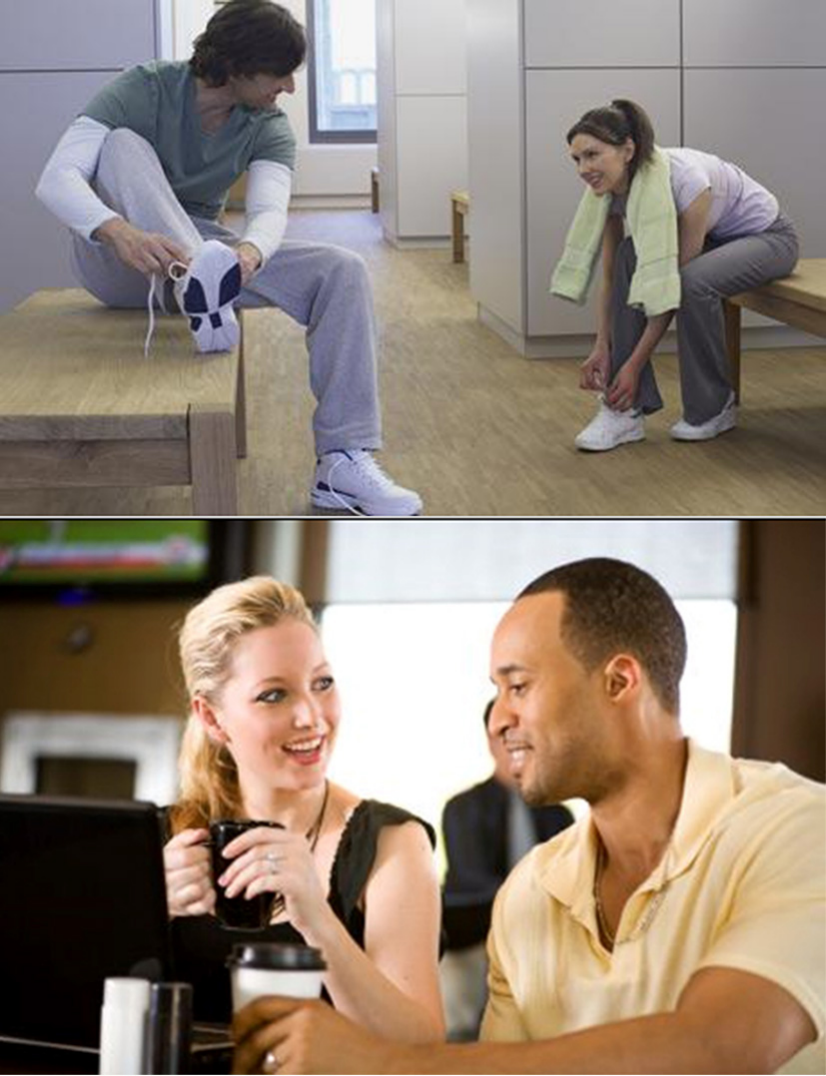
**Sample picture used in Experiment 2**.

The experiment was conducted in the morning (from 7:00 a.m. to noon). All participants were instructed not to eat for at least 6 h prior to the experiment. When the participants arrived at the laboratory, an initial check of their BG levels was performed. Their moods were assessed using a 9-point Likert scale ranging from extremely unhappy (1) to extremely happy (9). The participants were then asked to complete a photo rating task. In this task, the participants were asked to estimate the content and intent of a conversation between a man and a woman portrayed in a photo (see Figure 3 for examples). The participants were asked to rate the questions on a 5-point likelihood scale ranging from 1 (impossible) to 5 (definitely): “How likely is it that they are talking about work/flirting/gossiping about others/discussing politics?”

After completing the rating task, the participants were randomly assigned to either a water group in which they drank a 250 ml glass of water or a sugary drink group in which they drank a 230 ml glass of water with 45.4 g cane sugar. The participants were given a 10-min break after drinking. After the break, the participants underwent a second BG check and a second mood assessment. Finally, the participants were asked to complete a second photo rating task similar to the first task but with a different photo. The presentation order of the photos used in the two rating tasks was counterbalanced among the participants in the two drinking conditions.

### Results and discussion

**Figure 4:**
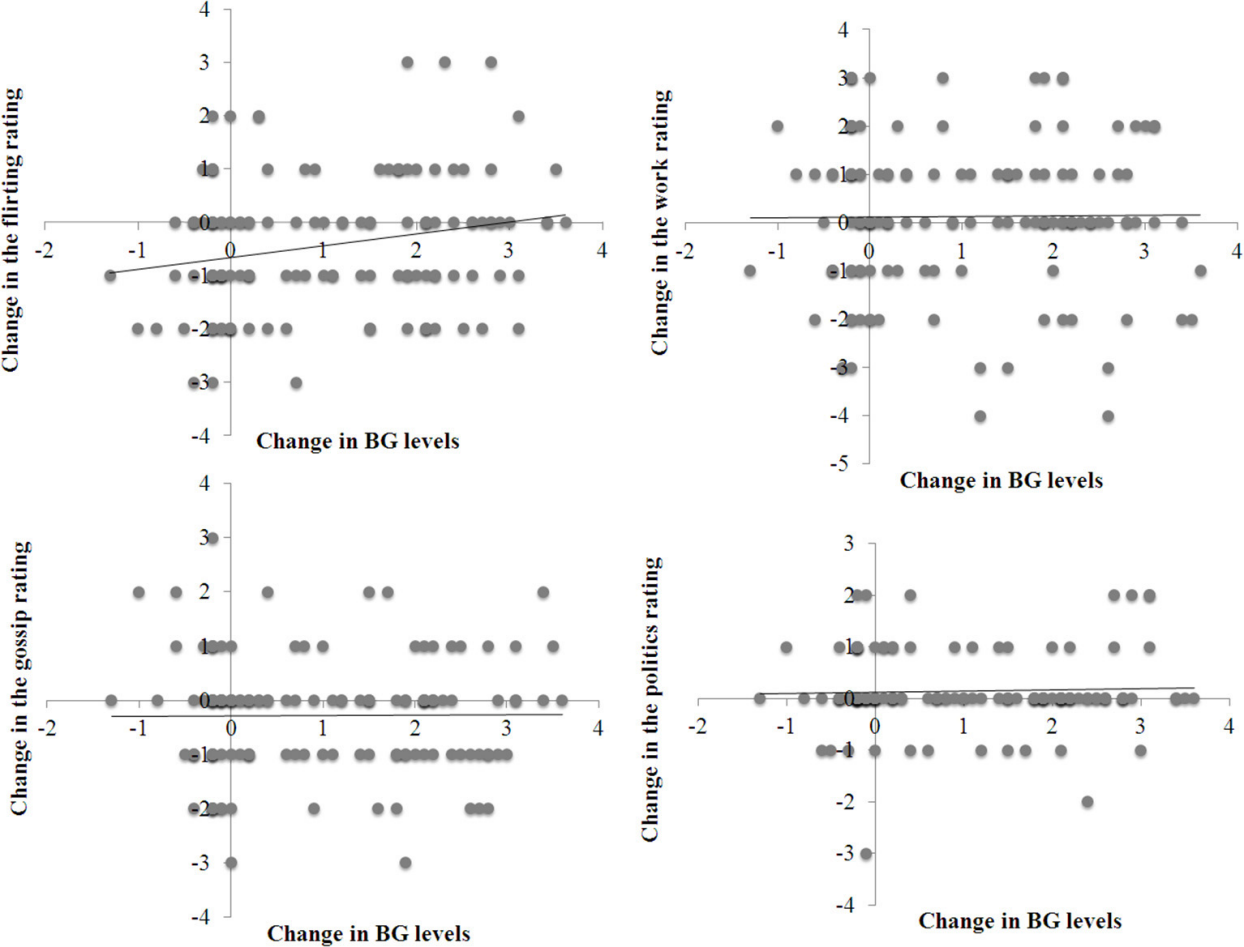
**Scatter plots of the change in four rating scores for the photos (the rating before drinking minus the rating after drinking) as a function of the change in blood glucose levels (the second BG check minus the first BG check)**.

Table 1 presents the descriptive results of Experiment 2. Figure 4 shows four scatter plots, one for each of the four photo ratings (i.e., the likelihood of flirting, gossiping, talking about work, or politics). Each point on a scatter plot represents the change in a rating score for one participant as a function of the change in his or her BG levels after drinking either the sugary drink or the water. For both the likelihood ratings and BG levels, a change score was the difference in the score after the drink minus the score before the drink.

**Table 1:** **The descriptive results of Experiment 2 (mean ± SE)**.

	**Blood sugar**	**Flirting**	**Work**	**Gossip**	**Politics**
Before drink	5.23 ± 0.04	2.73 ± 0.09	2.19 ± 0.08	2.78 ± 0.09	1.43 ± 0.06
Sugary drink	7.12 ± 0.09	2.35 ± 0.11	2.27 ± 0.12	2.57 ± 0.11	1.61 ± 0.08
Water drink	5.18 ± 0.05	2.23 ± 0.11	2.38 ± 0.14	2.43 ± 0.11	1.55 ± 0.09

To test the relationship between the change in BG levels and the change in photo interpretation ratings, we conducted four hierarchical multiple regression analyses of the four rating scores with the change in BG levels as the predictor variable. To control for other possible sources of effects on the rating scores, we entered the participants' gender, age, BMI, time of testing, mood change, and presentation order as covariates.

The regression analyses yielded a significant effect of BG changes on the flirting likelihood rating (Table 2). A change in BG levels was positively associated with a change in the subjective likelihood of flirting. None of the covariates were significant (Table 2). By contrast, the analyses revealed no significant effect of BG changes on other likelihood ratings (Table 2). The results of regression analyses were similar when the analysis excluded demographic variables (i.e., gender, age, BMI, time of testing, mood change).

**Table 2:** **The regression results of Experiment 2**.

	**Step 1**	**Step 2**	**Step 1**	**Step 2**	**Step 1**	**Step 2**	**Step 1**	**Step 2**
**STEP 1**								
Gender	−0.058	−0.079	0.043	0.044	0.145	0.150	0.107	0.101
Age	0.032	0.032	−0.038	−0.038	0.017	0.018	−0.126	−0.126
BMI	0.025	0.037	0.141	0.140	−0.107	−0.110	0.030	0.033
Time of testing	0.076	0.107	−0.129	−0.132	−0.031	−0.036	0.059	0.066
Mood change	−0.070	−0.098	0.157^*^	0.159^*^	0.082	0.088	−0.078	−0.085
Presentation order	−0.073	−0.067	−0.474^*^	−0.474^*^	0.263^*^	0.261^*^	−0.177^*^	−0.175^*^
**STEP 2**								
BG change		0.235^*^		−0.019		−0.042		0.053
*R^2^*	0.127	0.263	0.537	0.538	0.317	0.319	0.260	0.265
Δ*R^2^*		0.053^*^		0.001		0.002		0.003

* *denotes p ≤ 0.05*.

The results suggested a bidirectional relationship between food calories ingested and sensitivity to mating cues. That is, daily changes in BG levels within a normal range may sensitize or desensitize an individual to mating cues. The existing literature on attitudes and social perceptions provides strong confirmation that social judgment can be affected by environmental cues such as physical appearance (Livingston, 2001), geographical distance (Burgoon et al., 2013), and personal status (Pettit and Sivanathan, 2012). Our result suggests that when the body has a surplus in its food energy budget, as signaled by elevated BG levels, the mind becomes more sensitive to mating cues, whereas decreasing BG levels are associated with lower sensitivity to mating cues.

## General discussion

Within a synthetic framework of life-history theory and evolutionary tradeoffs of need prioritization, we explored a “food-mate” (survival-reproduction) tradeoff regulated by either external priming cues (Experiment 1) or internal metabolic cues of BG fluctuations (Experiment 2). In Experiment 1, mating opportunity cues promoted spending, whereas food availability cues promoted savings. This result suggests that investment decisions are regulated by external cues that indicate the availability of different types of resources (food or mates). In Experiment 2, we examined the same food-mate tradeoff regulated by BG fluctuations (see Figure 1).

Changes in the BG levels of the participants were positively associated with changes in the ratings of perceived flirtatiousness but were not associated with the ratings of other perceived intentions or activities. After drinking water, the participants in the fasting condition became less receptive to mating cues. Water drinking further lowered their BG levels and their perceptions of flirtatiousness. By contrast, although consumption of a sugary beverage was unable to reverse this trend, this consumption buffered the decline in sensitivity to mating cues. These results suggest that when one is hungry, the cognitive priority of mating decreases. In our results, the decrease in BG levels observed after drinking water had a significant effect on perceived sexual intentions, whereas the increasing BG levels after drinking a sugary beverage did not significantly increase receptivity to mating cues. Future studies should explore possible asymmetrical cognitive effects of caloric deficiency and caloric supplementation. According to life-history theory (Kaplan and Gangestad, 2005), tradeoffs among survival, growth, reproduction, and other needs are a function of their relative priorities in different stages of the life-span of an organism (Sibly and Calow, 1986). However, little is known about the need tradeoffs that occur on a daily basis. Our findings suggest that daily BG fluctuations serve as a signaling mechanism for regulating the cognitive priorities of different needs and motives.

Neurological evidence suggests similar mechanisms for different reinforcers also involve in need tradeoffs. For instance, the neural circuitry implicated in reward valuation in the nucleus accumbens is activated by either high-calorie food or sexual stimuli (Ikemoto and Panksepp, 1999). Similarly, the orbitofrontal cortex is activated by monetary rewards (Breiter et al., 2001), sweet-tasting food rewards, and the cues for such rewards (O'Doherty et al., 2002). These findings suggest that there is a neural basis for tradeoffs between physiological needs and mating needs.

In sum, our results suggest that the cognitive priority of each fundamental human need is dynamic and varies as a function of ongoing physiological conditions and resource availability in the environment.

### Conflict of interest statement

The authors declare that the research was conducted in the absence of any commercial or financial relationships that could be construed as a potential conflict of interest.

## Footnotes

^1^Similar mating cues were also used in previous studies (e.g., Mikulincer et al., 2001; Hundhammer and Mussweiler, 2012). These photos of romantic actions emphasize that mating cues not only are sexually provoking but also involve mutual attractions.
